# Supplementary material for: Chromothripsis during telomere crisis is independent of NHEJ, and consistent with a replicative origin
Source: Genome Res. 2019 May;29(5):737–49. doi: 10.1101/gr.240705.118 (PMC6499312; doi:10.1101/gr.240705.118)
Supplement: Supplemental Material [file supp_gr.240705.118_Supplemental_file_1.zip › contigs/annotated_contigs/DB93/contig.2.DB93_length_351_mean_cov_5.1566951567.docx]

**DB93_length_351_mean_cov_5.1566951567**

```
CAGAGTATAGATGGGAACACCCTCTACTTTGTACTCAATTTTGCTGTGAACCTAAAACTTCTCTGAAAGATAAATTTTATTTTGAAAAA
 >chr21:30264219-30264487 + E=4e-144
CACTATGATACACTAAAACTTCTTTTGTCCTTATTTGGTAATACTTAGAAATATAATTTTATTAAGATTATATTTAAAAGTAGAGTCAA

AAGATATGCATGTTTTAGTGAAATATCTTCTATTGTTATTACTATCATCATAGTTATTATTATTTGACTTAGAAATCCTTATTAGACAT

T|GTGAGAGTGTGTGTGAGTAAGTGTGTATGTGAGT|GTGAGTGAGACTGTATATATGTGCTGAGTAGGAGAGAGGGGGTCAGGGA   >chr3:81595386-81595420 - E=5e-03
```
